# Supplementary material for: Sources and Drivers of ARGs in Urban Streams in Atlanta, Georgia, USA
Source: Microorganisms. 2022 Sep 8;10(9):1804. doi: 10.3390/microorganisms10091804 (PMC9503305; doi:10.3390/microorganisms10091804)
Supplement: Supplementary file 1 [file microorganisms-10-01804-s001.zip › microorganisms-1829462-supplementary.pdf]

Sources and drivers of ARGs in Urban Streams in Atlanta, Georgia, USA

Supplementary material

Robert A. Sowah [1,2], Marirosa Molina [3,*], Ourania Georgacopoulos [4], Blake Snyder [2] and Mike Cyterski [5]

1 Oak Ridge Institute for Science and Education, Oak Ridge, TN 37831, USA
2 U.S. EPA, Laboratory of Services & Applied Sciences Division, 980 College Station Rd., Athens, GA 30605, USA
3 U.S. EPA, Office of Research and Development, Center for Environmental Measurement and Modeling,
109 T. W. Alexander Drive, Durham, NC 27709, USA
4 Student Services Contractor to the U.S. EPA, Office of Research and Development,
Center for Environmental Measurement and Modeling, 960 College Station Rd., Athens, GA 30605, USA
5 U.S. EPA, Office of Research and Development, Center for Environmental Measurement and Modeling,
960 College Station Rd., Athens, GA 30605, USA
* Correspondence: molina.marirosa@epa.gov

2. Methods

DNA extraction

Filters with concentrated microbial DNA were extracted using the DNeasy PowerLyzer PowerSoil kit (Qiagen, Germantown, MD). DNA extraction followed manufacturer's instructions with the following modifications: bead beating was performed at 6.5 m/s for 45 s with a Fastprep-24 Homogenizer (MP Biomedicals, Solon, OH); and only half of the bead solution and C1 mixture (405 μL) was transferred after the first step because it was difficult to carry over all of the supernatant accurately due to presence of the filter residue in the extract [41].

Development of qPCR standards

The development of plasmid DNA standards for EC23S857 (*E. coli* marker), HF183/BacR287 (HF183), *sulII*, *ampC* and *bla*SHV markers followed the procedures described in Oladeinde *et al*. [42] using genomic DNA from American Type Culture Collection (ATCC) and wastewater samples. Plasmid DNA standards were developed by amplifying sequence specific DNA targets using end-point PCR and target specific primers. Amplified DNA products were ligated into a pCR 2.1-TOPO plasmid vector, transformed into One Shot Top10 chemically competent *E. coli*, using a Topo TA kit (Life Technologies, Grand Island, NY) and plated on ImMedia ampicillin and kanamycin agar (Life Technologies). Recombinant bacteria colonies were selected and cultured overnight in ImMedia broth (Life Technologies). Plasmids were extracted using a PureLink Quick plasmid miniprep kit (Life Technologies) and then linearized with BamHI-HF enzyme (New England BioLabs, Ipswich, MA). Linearized plasmid DNA was purified using a QIAquick PCR purification kit (Qiagen Inc., Valencia, CA) and quantified with a NanoDrop ND-1000 UV/Vis spectrophotometer (NanoDrop Technologies). Plasmid DNA standards were serially diluted 10-fold to generate

qPCR standard curves for marker quantification. A synthesized gene fragment containing the target sequences for the genes *int1*, 16S rRNA, *sulI*, *tet*W and *tet*M was used to prepare standards [43].

qPCR assays

The primers and probes used in this study are detailed in Table S1. Plasmid DNA standards were serially diluted 10-fold to generate reaction standards. The dynamic range of standards varied between $10^2 – 10^8$ copies for the 16S rRNA marker and $10 – 10^6$ copies for all other markers. Each qPCR reaction mixture contained 10 ul of Taqman or SYBR Green Master Mix, optimized concentrations of forward and reverse primers, probe and 2 μl of sample DNA (in duplicate) in a final reaction volume of 20 μl. All qPCR reactions included 0.05 mg/ml of bovine serum albumin (BSA)(Life Technologies). Optimized primer concentrations were 0.9 μM for *E. coli* marker, HF183 and *sulI*, 1 μM for 16S rRNA, 0.3 μM for int 1, 0.5 μM for *tet*W and *tet*M, 0.15 μM for *ampC* and *bla*SHV, and 0.2 μM for *sulII*. For Taqman assays that require a probe, the following probe concentrations were used; 0.2 μM for *E. coli* marker, *sulI*, *tet*W and HF183, 0.5 μM for 16S rRNA, 0.25 μM for int1 and 0.25 μM for *tet*M. qPCR cycling conditions for Taqman assays consisted of a 20 s hold at 95°C, followed by 40 cycles of 95°C for 5 s and 60°C for 30 s. The cycling conditions for SYBR Green assays consisted of a hold step of 10 min at 95°C followed by 95°C for 15 s and a final step of 60°C for 30 s.

qPCR inhibition test and lower limit of quantification (LLOQ)

The presence of humic acids and other organic substances in environmental samples have been reported to inhibit PCR [44,45]. A test for qPCR inhibition in sample DNA was performed using salmon testes DNA and Sketa22 marker as previously described [41]. Briefly, 4 ul of sample DNA was added to a qPCR reaction consisting of Sketa22 primers and probe, BSA, and 0.05 ng/ul of salmon testes DNA (Sigma, St. Louis, MO). Mean uninhibited salmon C_T values were obtained by adding salmon DNA to duplicate control samples of method blanks. Reactions were deemed inhibited if the salmon C_T value was 2 units higher than the average salmon C_T observed for the control samples. All samples analyzed did not show inhibition. The LLOQ for qPCR assays was determined as the lowest copy number of the qPCR standards that was accurately measured in all assays [29 – 32]. Therefore, the lowest copy number of the standards within the linear range of quantification was taken as the assay LLOQ which also represents the limit of detection in this study. The LLOQ was 100 copies/2μl for the 16S rRNA and 10 copies/2μl for all other markers.

Quality control for qPCR

Melt-curve analysis was performed for SYBR Green reactions to distinguish specific PCR products from non-specific products. All qPCR runs also included positive controls (plasmid DNA), negative controls (in triplicate) containing nuclease free water, method blanks and field blanks. Standard curves, which were calculated as simple linear regressions, were used to calculate amplification efficiencies (which ranged from 90 to 110% in this study) for each instrument run. All standard curves generated for marker quantification in this study had a goodness of fit R^2 value above 0.98.

Data analysis

Generalized Boosted Models

For an in-depth discussion of these parameters, see the GBM package documentation [46]. Recommendations within Greenwell *et al*. [46] were followed for setting parameter values, with some modifications based on best professional judgement. Train Fraction was set to 1 because a true testing dataset was created at the start of each iteration for assessment of model predictive capabilities. In addition, evaluation of out-of-sample error would be handled by examining cross-validation folds, as explained later. A Bag Fraction of 0.5 results in each successive tree in the iterative GBM algorithm being fit to a random 50% of the observations in the training dataset, which mitigates overfitting. An Interaction Depth of 3 means that up to third-order interactions of model covariates can be captured by the eventual solution forest. We did not include interactions of an order greater than 3 to preserve model interpretability. The Minimum Number of Observations per Node value prevents the model from being unduly influenced by single outliers or small clusters of odd samples. A value of 5 was deemed suitable for this intermediate-sized dataset (n between 100 and 1000). Smaller values of the Shrinkage parameter can increase model accuracy, but at the cost of increased computational time and more trees in the optimal solution. Values between 0.01 and 0.001 are recommended; we used 0.005.

As more trees are added to a GBM solution, the training data error (RMSE) will continue to decline; the RMSE of out-of-sample data initially declines as more trees are added, but then rises if too many are used, i.e., the model becomes overfit. There are several ways to determine the optimum number of trees for the final GBM solution; we used 10-fold cross-validation to measure the point at which out-of-sample RMSE began to rise. Due to the stochasticity in creating training/testing datasets, a Bag Fraction < 1, and the random splitting of the training data into cross-validation folds, sometimes a GBM model can produce a poor solution. In each iteration of the bootstrap process, we used two metrics to ensure a GBM model was "valid:"

- The optimum solution had less than 10000 trees (maximum allowable, meaning convergence was achieved)
- The number of unique fitted values produced by the model was at least 25% of the total number of fitted values

One hallmark of a poorly-fit GBM model is a solution with few trees, leading to a very low number of unique fitted values. However, it is also possible to sometimes reach a good solution with a relatively small number of trees. Therefore, it is more robust to assess the quality of the model by examining the number of unique fitted values than the number of trees in the solution.

The bootstrap algorithm was run until 500 "valid" GBM models were found, and then we examined their characteristics:

- R^2 of Actual Observations versus Model Fits for the Training Data

- R^2 of Actual Observations versus Model Fits for the Testing Data
- The Influence of the Covariates

Table S1. Primers and Probes used in this study

Assay Name	Primer	Sequence 5' - 3'	Amplicon Size (bp)	Reference
sulII	*sulII* - F	TCCGGTGGAGGCCGGTATCTGG	191	Pei *et al* 2006
	sulII - R	CGGGAATGCCATCTGCCTTGAG		
sulI	*sulI* - F	CCGTTGGCCTTCCTGTAAAG	67	Calero-Caceres *et al.*, 2014
	sulI - R	TTGCCGATCGCGTGAAGT		
	sulI - P	FAM-CGAGCCTTGCGGCGG-MGBNFQ		
*tet*W	*tet*W - F	CGGCAGCGCAAAGAGAAC	58	Walsh *et al.*, 2011
	*tet*W - R	CGGGTCAGTATCCGCAAGTT		
	*tet*W - P	FAM-CTGGACGCTCTTACG-MGBNFQ		
*tet*M	*tet*M - F	GGTTTCTCTTGGATACTTAAATCAATCR	67	Peak *et al.*, 2007
	*tet*M - R	CCAACCATAYAATCCTTGTTCRC		
	*tet*M - P	FAM-ATGCAGTTATGGARGGGATACGCT ATGGY-BHQ1		
ampC	*ampC* - F	TGAGTTAGGTTCGGTCAGCA	98	Fernando *et al* 2016
	ampC - R	AGTATTTTGTTGCGGGATCG		
*bla*SHV	*bla*SHV - F	CGCTTTCCCATGATGAGCACCTTT	110	Xi *et al* 2009
	*bla*SHV - R	TCCTGCTGGCGATAGTGGATCTTT		
int1	*int1* - F	GCCTTGATGTTACCCGAGAG	196	Barraud *et al.* 2010
	int1 - R	GATCGGTCGAATGCGTGT		
	int1 - P	FAM- ATTCCTGGCCGTGGTTCTGGGTTTT -BHQ1		
HF183/Bac R287	HF183 - F	ATCATGAGTTCACATGTCCG	126	Green *et al.* 2014
	BacR287 - R	CTTCCTCTCAGAACCCCTATCC		
	BacP234MGB - P	FAM-CTAATGGAACGCATCCC-MGB		
EC23S857	EC23S858 - F	GGTAGAGCACTGTTTTGGCA	88	Chern *et al.* 2011
	EC23S858 - R	TGTCTCCCGTGATAACTTTCTC		
	EC23S858 - P	FAM-TCATCCCGACTTACCAACCCG-TAMRA		
16S rRNA	BACT1369F	CGGTGAATACGTTCYCGG	173	Suzuki *et al.* 2000
	PROK1541R	AAGGAGGTGATCCRGCCGCA		
	TM1389F	FAM-CTTGTACACACCGCCCG-BHQ1		
Sketa22	SketaF2	GGTTTCCGCAGCTGGG		Haugland *et al.*, 2005
	SketaR3	CCGAGCCGTCCTGGTCTA		
	SketaP2	FAM-AGTCGCAGGCGGCCACCGT-TAMRA		

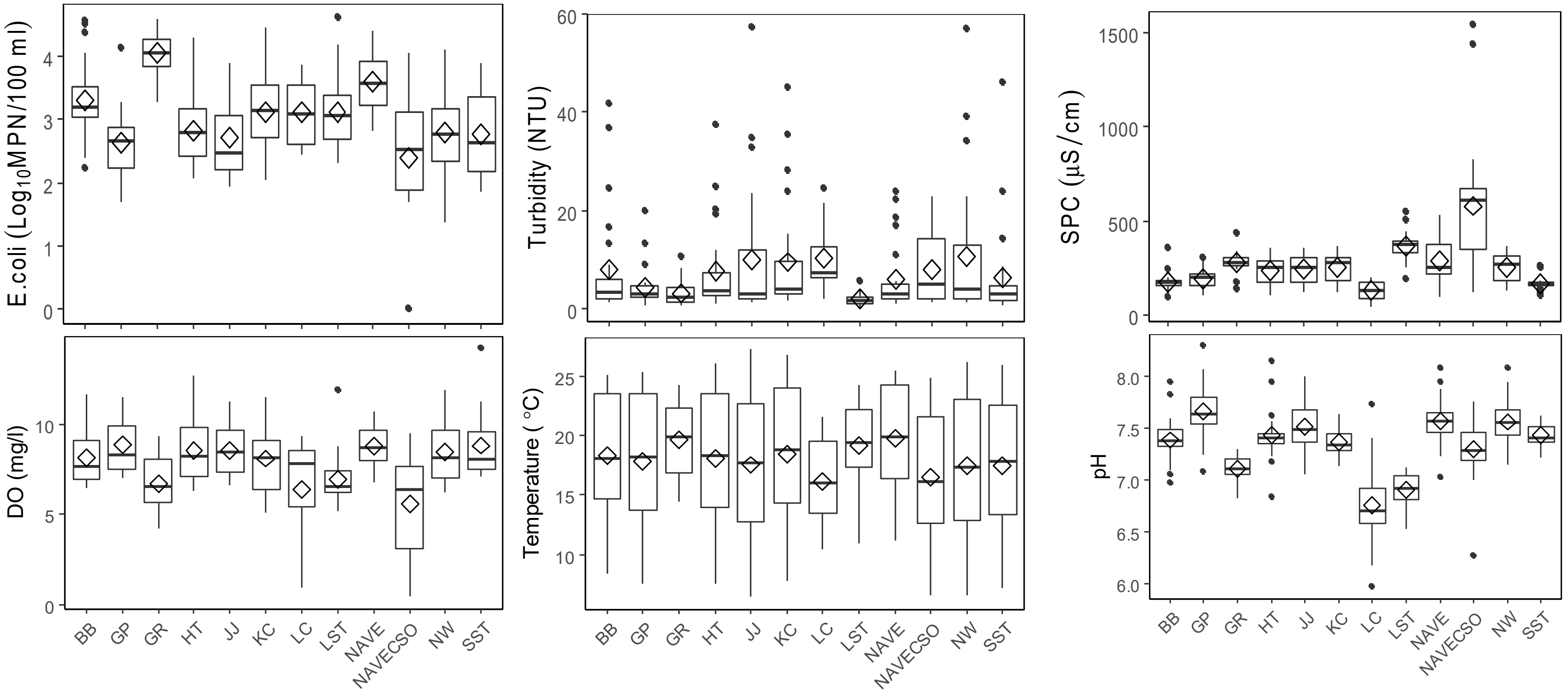

Figure S1. Distribution of standard water quality parameters including *E. coli*, SPC (specific conductance), Water Temperature, Turbidity, pH and DO (dissolved oxygen). The boxplots show the 25th quartile , 75th quartile and median lines. The diamond shape represents the mean observations for each site, whilst outliers are represented by the solid points.

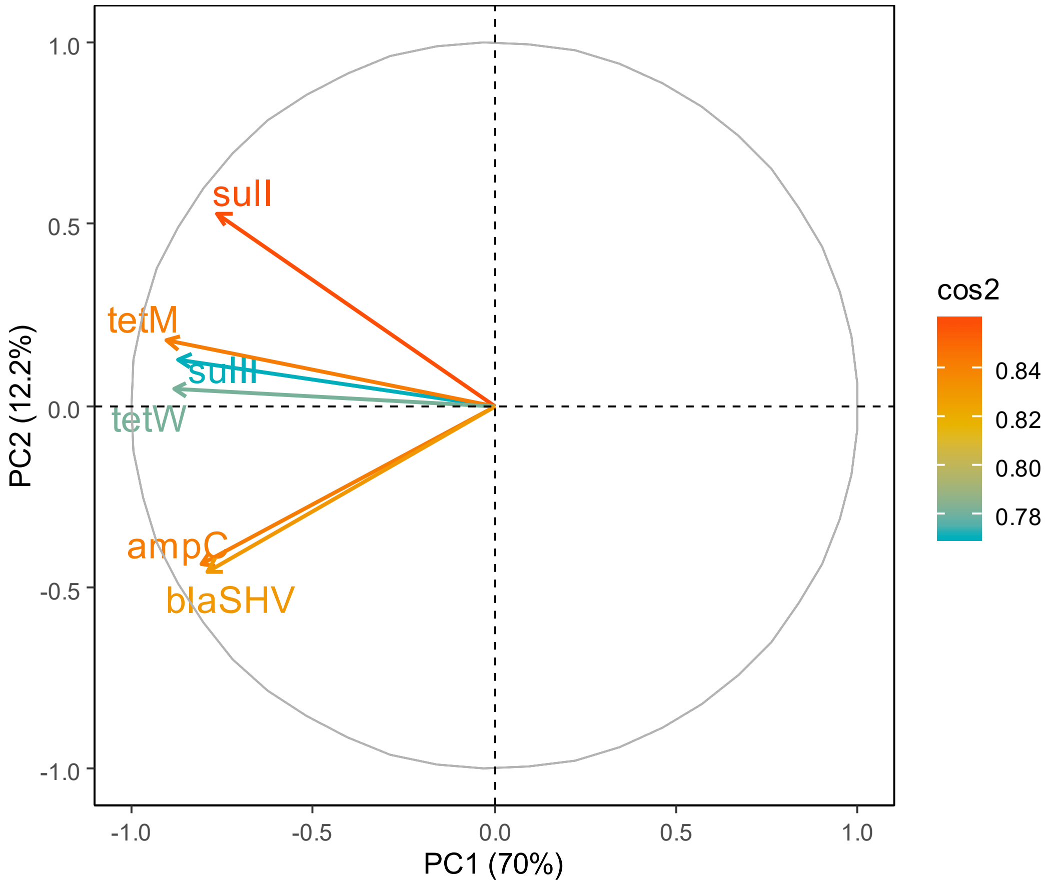

Figure S2. Principal component plot showing the loading of ARGs and the influence on first two principal components, PC1 and PC2. The color gradient shows the quality of representation of the ARGs on the principal components
